# Species-Specific Immunity Induced by Infection with *Entamoeba histolytica* and *Entamoeba moshkovskii* in Mice

**DOI:** 10.1371/journal.pone.0082025

**Published:** 2013-11-29

**Authors:** Chikako Shimokawa, Richard Culleton, Takashi Imai, Kazutomo Suzue, Makoto Hirai, Tomoyo Taniguchi, Seiki Kobayashi, Hajime Hisaeda, Shinjiro Hamano

**Affiliations:** 1 Department of Parasitology, Nagasaki University, Nagasaki, Japan; 2 Malaria Unit, Institute of Tropical Medicine (NEKKEN), Nagasaki University, Nagasaki, Japan; 3 Department of Parasitology, Graduate School of Medicine, Gunma University, Gunma, Japan; 4 Department of Tropical Medicine and Parasitology, Keio University School of Medicine, Shinjuku, Tokyo, Japan; 5 Global COE Program, Nagasaki University, Nagasaki, Japan; Obihiro University of Agriculture and Veterinary Medicine, Japan

## Abstract

*Entamoeba histolytica*, the parasitic amoeba responsible for amoebiasis, causes approximately 100,000 deaths every year. There is currently no vaccine against this parasite. We have previously shown that intracecal inoculation of *E. histolytica* trophozoites leads to chronic and non-healing cecitis in mice. *Entamoeba moshkovskii*, a closely related amoeba, also causes diarrhea and other intestinal disorders in this model. Here, we investigated the effect of infection followed by drug-cure of these species on the induction of immunity against homologous or heterologous species challenge. Mice were infected with *E. histolytica* or *E. moshkovskii* and treated with metronidazole 14 days later. Re-challenge with *E. histolytica* or *E*. *moshkovskii* was conducted seven or 28 days following confirmation of the clearance of amoebae, and the degree of protection compared to non-exposed control mice was evaluated. We show that primary infection with these amoebae induces a species-specific immune response which protects against challenge with the homologous, but not a heterologous species. These findings pave the way, therefore, for the identification of novel amoebae antigens that may become the targets of vaccines and provide a useful platform to investigate host protective immunity to *Entamoeba* infections.

## Introduction

Amoebiasis, an infectious disease caused by the parasitic protozoan *Entamoeba histolytica* is responsible for over 50 million cases in tropical regions and nearly 100,000 deaths worldwide each year. Infection is initiated through the ingestion of cysts in contaminated food or water. *E. histolytica* primarily infects the intestine, and may cause a wide range of symptoms from mild diarrhea to serious dysentery. If untreated, the parasite can cause life-threatening hemorrhagic colitis and/or extra-intestinal abscesses [1-5].

*E. histolytica* trophozoites are able to colonize the human intestine by adhering to colonic mucins and subsequently to epithelial cells via cell surface lectin [6]. This lectin is important for colonic colonization by *E. histolytica*. A colonization-blocking vaccine targeting this parasite lectin could prevent trophozoite adherence and thus provide protection against subsequent invasive disease [7]. Furthermore, recently, it has also been reported that there is a correlation between the presence of anti-lectin fecal immunoglobulin A (IgA) antibodies and protection from parasitic colonization in humans and mice [7-9]. These reports suggest that amoebiasis can be controlled by acquired immunity.

*Entamoeba moshkovskii* is closely related to *Entamoeba dispar* and *E. histolytica* and is microscopically indistinguishable from them in its cyst and trophozoite forms [10]. Recently, we reported that *E. moshkovskii* causes diarrhea, colitis and weight loss in mice, and that in Bangladeshi children, acquisition of *E. moshkovskii* infection was associated with diarrhea [11].

Here, using *E. histolytica* and *E. moshkovskii* infections in mice, we evaluate whether the immunity against reinfection that occurs following a primary infection is species-specific. We find that, following a primary infection with either *E. histolytica* or *E. moshkovskii*, mice are protected from re-challenge with a homologous species, but remained susceptible to a heterologous species. These results show, for the first time, that the immunity acquired during primary infection with *Entamoeba* spp. confers species-specific protective immunity.

## Materials and Methods

### Mice

Male CBA/J mice were purchased from Jackson Laboratories. Animals were maintained under specific pathogen free conditions at the Animal Research Center for Tropical Infectious Diseases, Nagasaki University, and were challenged when they were 5-8 weeks old. All experiments that involved mice were reviewed and approved by the Committee for Ethics on Animal Experiments of the Graduate School of Nagasaki University, and were conducted under the control of the Guidelines for Animal Experiments in the Graduate School of Medicine, Nagasaki University, and the Law (No. 105) and Notification (No. 6) of the Japanese Government pertaining to the use of experimental animals.

### Parasite Culture and Infection

Trophozoites of *E. histolytica*, originally laboratory strain HM1:IMSS (American Type Culture Collection, Manassas, VA), were from Prof. Eric Houpt, University of Virginia, and were serially passaged *in vivo* through the ceca of mice [12]. Trophozoites of the *E. moshkovskii* Laredo strain, were a gift from Dr. Seiki Kobayashi, Keio University, School of Medicine (originally from the late professor Louis S. Diamond, NIH, Bethesda, Maryland). Cecal contents were cultured at 37°C and 25°C, respectively, in BIS-33 medium supplemented with heat-inactivated 10% adult bovine serum, 25U/ml penicillin and 25 mg/ml streptomycin [13]. Trophozoites in the logarithmic growth phase were used in the experiments.

### Intracecal inoculation of *Entamoeba* spp

Trophozoites of *E. histolytica* HM1:IMSS and *E. moshkovskii* Laredo strain were collected after incubating the tubes on ice for 5-10 minutes. Then, the number of trophozoites was counted. We anesthetized mice with Domitor (medetomidine hydrochloride: 0.1mg/kg) and Dormicum (midazolam: 0.1 mg/kg), shaved their abdomens to incise the skin, exteriorized each cecum from the peritoneum, and injected 150μl of 1×10^6^ trophozoites into the apical sites of cecum. Then, the cecum was blotted and the peritoneum and the skin were sutured. Mice were kept on warming blankets at 37°C throughout surgery. Survival rates were ≥90% in all mice.

### Detection of each *Entamoeba* spp. by PCR using DNA extracted from stool of mice

For isolation of *Entamoeba* DNA from mouse stools, QIAamp DNA Stool Kits (QIAGEN, Valencia CA) were used according to manufacturer’s instructions. The primer sequences used for PCR are as previously described [14].

### Administration of metronidazole

For *in vivo* studies, stock solutions of metronidazole (Sigma Aldrich, St. Louis, MO) were prepared in 100% dimethyl sulfoxide at a concentration of 10 mg/mL and stored at 4°C. The stock solution was diluted 32 times with distilled water to 0.3125 mg/mL, in which the concentration of DMSO was 3.125%. Mice were treated orally with metronidazole at a dose of 12.5mg/kg of body weight. To cure primary infections with *E. histolytica* or with *E. moshkovskii*, all of mice challenged with *E. histolytica* or with *E. moshkovskii* were treated with 1 mL of metronidazole orally (0.3125 mg/mL) using gastric intubation on day 14 post-infection. Naïve mice were also administered with metronidazole and used as control.

### Statistical analysis

Differences between groups were analyzed for statistical significance with unpaired Student’s *t*-test and χ^2^ test. All of these were performed using Excel software. Probabilities below 0.05 were considered statistically significant.

## Results

### E. *moshkovskii* infections were resolved earlier than *E. histolytica* infections

**Figure 1:**
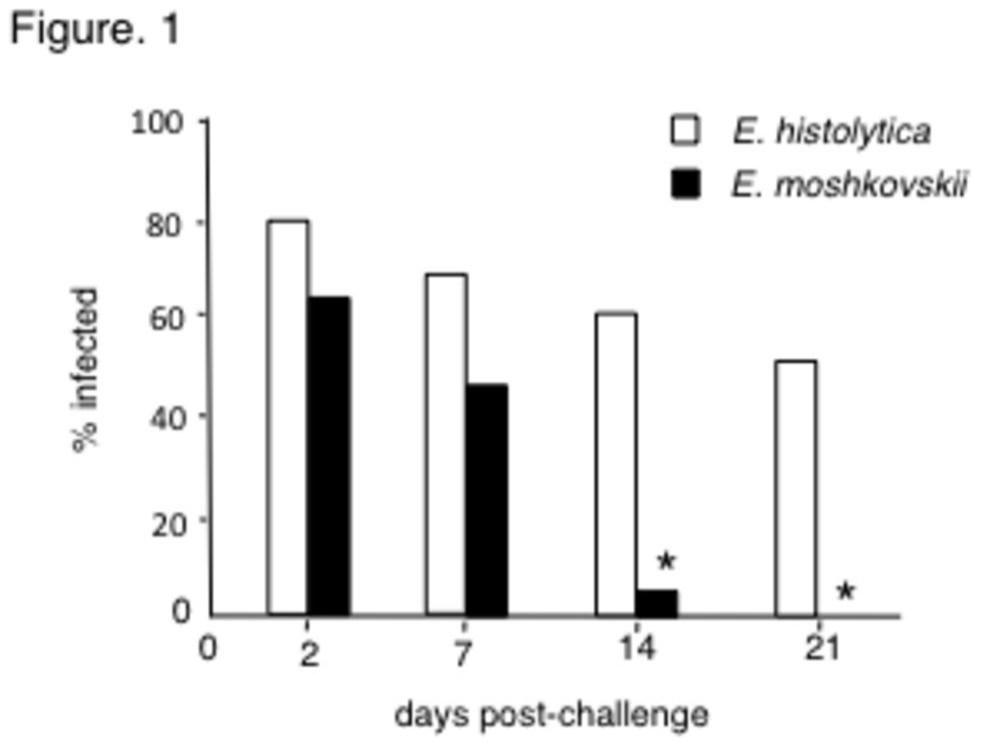
Entamoeba moshkovskii infections were resolved earlier than *Entamoeba histolytica* infections. CBA/J mice were intracaecally infected with 1 × 10^6^ trophozoites of *E. histolytica* and *E. moshkovskii*. Infection rate was monitored by detecting amoebae in caecal content and by amplifying the amoeba gene from faecal DNA on days 2, 7, 14, and 21. Infection rate of mice with *E. histolytica* and *E. moshkovskii* was shown as open and closed columns, respectively. Values show the representative result out of 3 individual experiments. Asterisks indicate statistical significance with p < 0.05 between mice infected with *E. histolytica* and *E. moshkovskii* by χ^2^ test.

We have previously demonstrated that C3H/HeN, C3H/HeJ and CBA/J mice allow the establishment of *E. histolytica* and *E. moshkovskii* infections, while many strains of mice including C57BL/6 and BALB/c mice do not, indicating that susceptibility to *E. histolytica* and *E. moshkovskii* infection is dependent on the genetic background of the host [11,12,15-17]. Trophozoites of *E. histolytica* and *E. moshkovskii* were intracecally inoculated into CBA/J mice. As expected, both *E. histolytica* and *E. moshkovskii* succeeded in infecting CBA/J mice after challenge (Figure 1). *E. histolytica* infected the ceca in approximately 80% of CBA/J mice (16 of 20) as confirmed by both culture and PCR of intracecal contents two days after challenge. In contrast, *E. moshkovskii* infected the ceca of CBA/J mice in approximately 65% of mice (13 of 20) at the same point. At day 14 post-challenge, the infection rate of *E. histolytica* was approximately 60% (12 of 20 mice positive), though that of *E. moshkovskii* was approximately 5% of mice (1 of 20). At 21 days post-challenge, the infection rate of *E. histolytica* was approximately 58% (11 of 19) and that of *E. moshkovskii* was 0%.

### Metronidazole Sensitivity in E. *moshkovskii*

**Figure 2:**
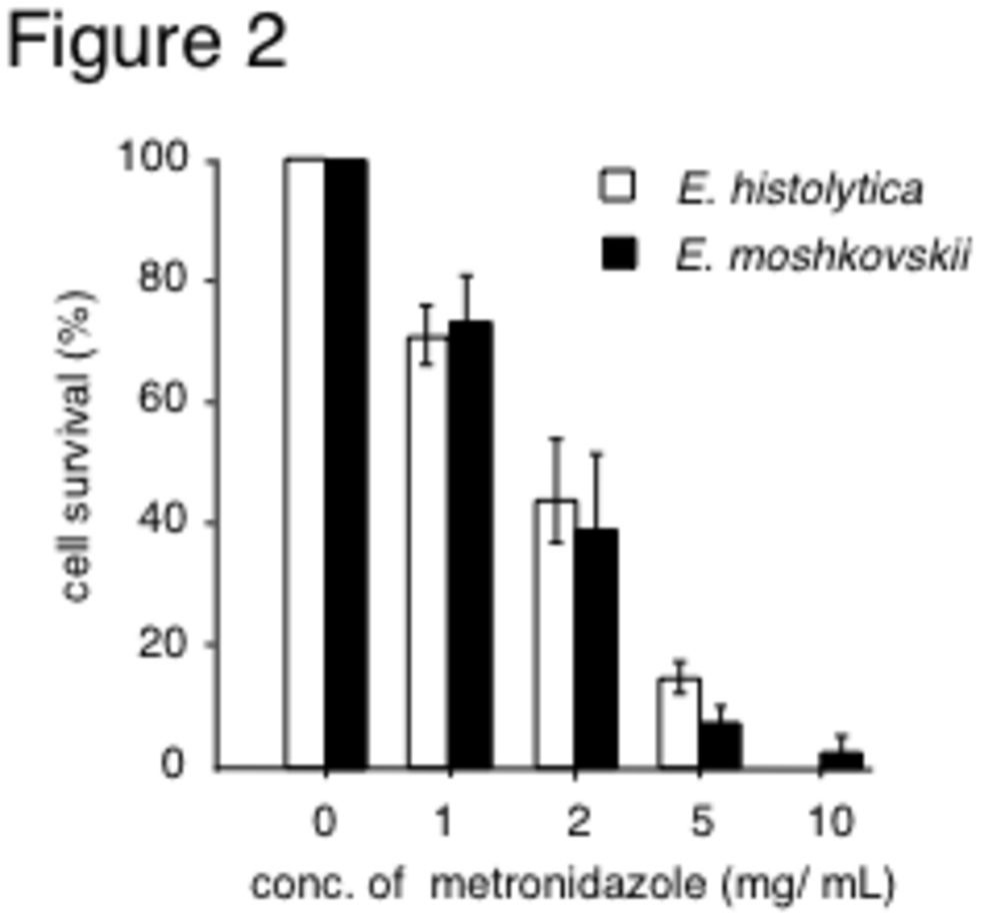
The effect of metronidazole on the growth and survival of *Entamoeba histolytica* and *Entamoeba moshkovskii* trophozoites was evaluated *in* *vitro*. *E. histolytica* and *E. moshkovskii* were incubated with various concentrations of metronidazole for 48h. Then, the number of viable cells was counted and the proportion of it versus initial number was shown as open and closed columns, respectively.

So as to treat mice infected with *E. histolytica* and *E. moshkovskii*, the effect of metronidazole on the growth and survival of *E. histolytica* and *E. moshkovskii* trophozoites was evaluated *in vitro*. The number of viable cells in glass tubes was counted after incubation of *E. histolytica* and *E. moshkovskii* with various concentrations of metronidazole for 48h. The numbers of *E. histolytica* and *E. moshkovskii* treated with metronidazole decreased significantly in a dose dependent manner (Figure 2).

### Amoebic infection induced species-specific protective immunity

**Figure 3:**
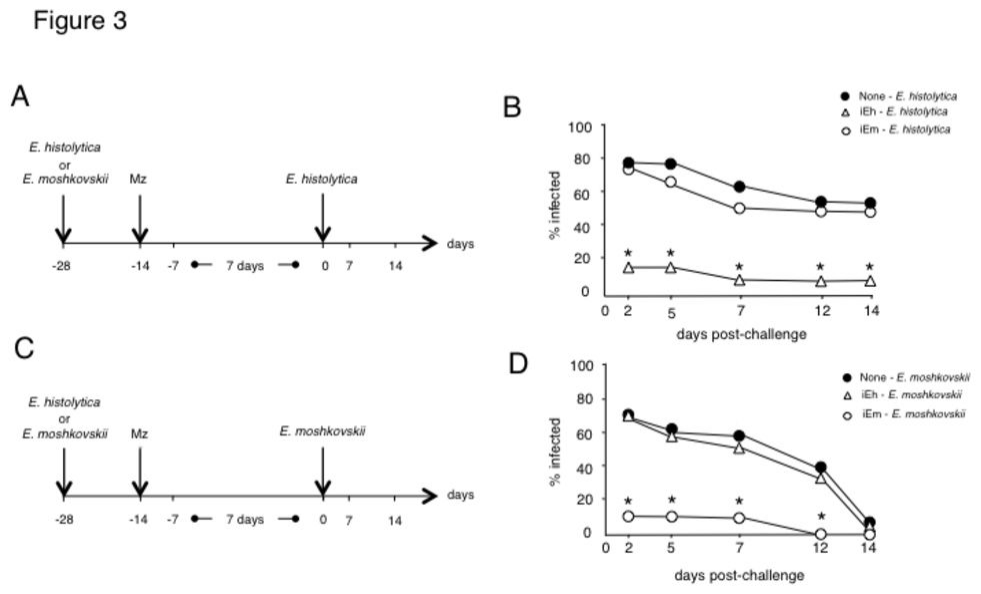
Amoebic infection induced species-specific protective immunity. Mice were infected with 1 × 10^6^ trophozoites of *Entamoeba histolytica* (open triangle) or *Entamoeba moshkovskii* (open circle) and treated with metronidazole (Mz) on day 14 following induction of the primary infection. The clearance of amoeba was confirmed seven days after treatment by PCR. Mice were kept without any intervention for an additional week, and then re-challenged with 1 × 10^6^ trophozoites of *E. histolytica* (A, B) or *E. moshkovskii* (C, D) at 14 days after treatment. The number of mice used was as follows: for naïve→*E. histolytica*, N=16: for *E. histolytica*→*E. histolytica*, N=20: for *E. moshkovskii *→*E. histolytica*, N=20 (A, B); for naïve→*E. moshkovskii*, N=20: for *E. histolytica*→*E. moshkovskii*, N=20: for *E. moshkovskii *→*E. moshkovskii*, N=20 (C, D). Asterisks indicate statistical significance with p < 0.05 by χ^2^ test between mice infected with *E. histolytica* and *E. moshkovskii* in the primary infection.

In order to examine whether protection against re-infection can be induced by primary infection, the mice that allowed the establishment of the primary infection with *E. histolytica* or *E. moshkovskii* were treated with metronidazole on day 14 and used for the secondary challenge. The clearance of amoeba was confirmed seven days after treatment by PCR. Mice were kept without any intervention for an additional week, and then re-challenged with *E. histolytica* or *E. moshkovskii* a total of 14 days after treatment (Figure 3A, C). The mice infected with *E. histolytica* and treated with metronidazole showed resistance to homologous re-challenge infection (Figure 3B), but allowed establishment of infection with the heterologous species *E. moshkovskii* in a manner similar to that seen in naive mice (Figure 3D). Similarly, mice infected with *E. moshkovskii* and treated with metronidazole showed resistance to homologous re-challenge infection with *E. moshkovskii* (Figure 3D), but allowed the establishment of infection with the heterologous species *E. histolytica* (Figure 3B). Thus, mice that experienced primary amoebic infection acquired resistance to secondary homologous species infection. However, primary amoebic infection did not confer protection against heterologous species secondary infection. These results show that intestinal amoebic infection induces species-specific protective immunity.

### The protection induced by primary infection lasts more than four weeks

**Figure 4:**
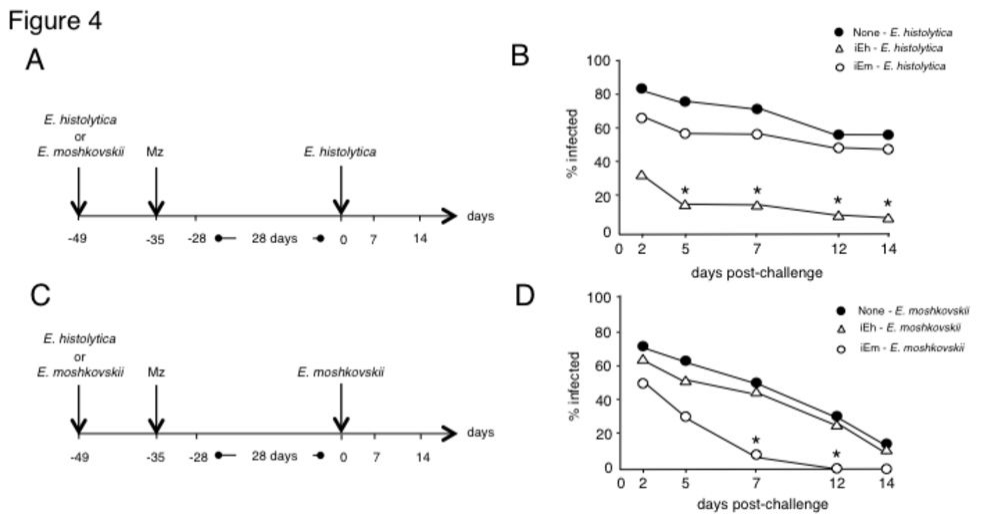
The protection induced by primary infection lasts more than four weeks. Mice were infected with 1 × 10^6^ trophozoites of *Entamoeba histolytica* (open triangle) or *Entamoeba moshkovskii* (open circle) and treated with metronidazole (Mz) on day 14 following induction of the primary infection. The clearance of amoeba was confirmed seven days after treatment by PCR. Mice were kept without any intervention for 28 days, and re-challenged with 1 × 10^6^ trophozoites of *E. histolytica* (A, B) or *E. moshkovskii* (C, D) at 35 days after treatment. The number of mice used was as follows: for naïve→*E. histolytica*, N=16: for *E. histolytica*→*E. histolytica*, N=20: for *E. moshkovskii *→*E. histolytica*, N=26 (A, B); for naïve→*E. moshkovskii*, N=15: for *E. histolytica*→*E. moshkovskii*, N=20: for *E. moshkovskii *→*E. moshkovskii*, N=20 (C, D). Asterisks indicate statistical significance with p < 0.05 by χ^2^ test between mice infected with *E. histolytica* and *E. moshkovskii* in the primary infection.

To examine how long the protection observed against secondary infection lasts, mice were re-challenged with homologous or heterologous amoebae on day 35 after treatment with metronidazole 14 days after primary infection. The clearance of amoeba was confirmed seven days after treatment by PCR (Figure 4A, C). As shown in Figure 4B and 4D, 35 days after the treatment of the primary infection, mice were resistant to homologous re-challenge, but were susceptible to heterologous species infection. Mice kept for 35 days after the treatment of the primary infection showed increased infection rates compared to those kept just for 14 days, a phenomenon that was most apparent on day 2 post re-challenge in the case of *E. histolytica* and on days 2 and 5 post challenge with *E. moshkovskii* (Figure 4B and 4D). These results suggest that the protection induced by primary infection may include not only memory responses but also remaining primary immune responses, both of which are species specific.

### Infection-induced species-specific immunity protects mice from weight loss

**Figure 5:**
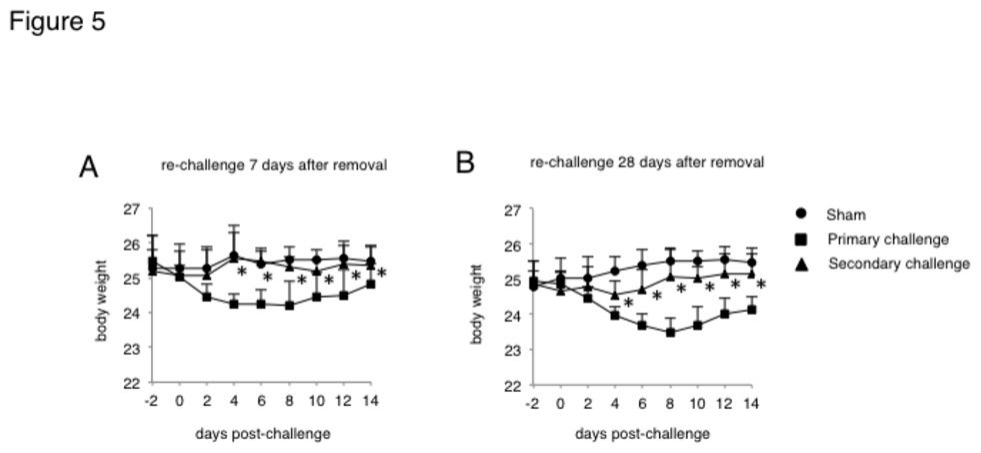
The change of body weight was monitored after re-challenging mice with *Entamoeba moshkovskii*. The naïve mice or mice exposed to primary *E. moshkovskii* infection were re-challenged with 1 × 10^6^ trophozoites of *E. moshkovskii* at 7 or 28 days after confirming the clearance of primary infection. The studies were repeated 3 times with similar results. Asterisks indicate statistical significance with p < 0.05 between the groups of mice with primary and secondary infection using unpaired Student’s *t*-test.

During the primary infection, mice infected with *E. moshkovskii* suffered severe symptoms. Following re-challenge with *E. moshkovskii* (homologous species) 14 days after treatment of the primary infection, mice did not show any weight loss (Figure 5A). Slight weight loss was observed, however, in mice re-challenged 35 days after treatment, but the severity of weight loss was much smaller than that observed during the primary infection (Figure 5B). The weight loss was also ameliorated in mice re-infected with *E. histolytica*, when having been given a primary infection with the homologous species (data not shown).

## Discussion

Our results clearly demonstrate that exposure to, and subsequent drug clearance of, the parasitic amoebae *E. histolytica* and *E. moshkovskii* invokes a strong immune response that protects mice from subsequent infection with a homologous species. This protection is species specific, affording little to no protection against a heterologous species challenge. The fact that this strong species-specific immunity was shown to last at least 35 days following the treatment of the initial infection suggests that memory responses are involved.

 Despite the fact that it has long been suspected that people may acquire immunity against amoebae, as older children in endemic areas are infected less frequently than younger children [18], this work constitutes the first experimental proof of this phenomenon. The molecular and cellular mechanisms responsible for the observed protection have not been addressed in this work. Mucosal IgA has been reported to be associated with protection against intestinal amoebiasis in humans, mice and baboons [7,8,19]. Indeed, monoclonal IgA specific for the *E. histolytica* galactose inhibitable adherence (GalNAc) lectin heavy subunit (HgL) is thought to inhibit its interaction with a host sugar moiety in colonic mucins, resulting in the failure of amoebae to settle within the intestines [20]. Furthermore, we and Guo et al. recently reported that IFN-γ derived from amoeba-specific T cells plays a protective role against *E. moshkovskii* (unpublished data) and *E. histolytica* [21], respectively, suggesting that T cells as well as antibodies specific for amoebic antigens are involved in acquired resistance to intestinal amoebic infections.

The phenomenon of species-specific immunity against parasitic pathogens has been studied in a number of parasitic species, perhaps most comprehensively with the *Plasmodium* species responsible for malaria [22]. For this pathogen, which exhibits both species and strain specific immunity, antigenic variation of major parasite surface antigens such as the merozoite surface protein 1 (MSP1), induces antibody-mediated immune responses that are effective only against the inducing-strain [22,23]. Such highly polymorphic strain- and species-specific antigens are thought to evolve through the actions of positive diversifying selection, so that proteins that are targeted by the host immune response rapidly accumulate polymorphisms. Here we show, for the first time, that the phenomenon of species-specific immunity also exists for *Entamoeba* *spp*. It seems probable that this is due to polymorphisms in major antigen target proteins between species. If so, then such antigens may be identified by comparative genomics. Of particular interest are the GalNAc-lectin HgL proteins previously implicated in antibody-mediated protection against *E. histolytica*. Nucleotide sequence comparisons of the genes encoding this protein in *E histolytica* and *E. moshkovskii* may shed further light on this.

 We found that *E. moshkovskii* is susceptible to the anti-amoeba drug metronidazole both *in vitro* and *in vivo* to the same degree as *E. histolytica*. This finding supports the use of this drug in the treatment of pathogenic *E. moshkovskii*, and may ease concern of treatment failure following cases of misdiagnosis of *E. moshkovskii* as *E. histolytica*.

 In summary, we show that exposure to a single drug cured amoebic infection confers resistance to re-challenge with the homologous, but not a heterologous species, for the first time, in which species-specific acquired immunity has been demonstrated for amoebic infections. This work paves the way, therefore, for the identification of novel amoebae antigens that may become the targets of vaccines.
