# Correction: Species-Specific Immunity Induced by Infection with *Entamoeba histolytica* and *Entamoeba moshkovskii* in Mice

**DOI:** 10.1371/annotation/aed3d4eb-ad0e-49d6-a5e7-6df2dcd7e3e7

**Published:** 2013-12-16

**Authors:** Chikako Shimokawa, Richard Culleton, Takashi Imai, Kazutomo Suzue, Makoto Hirai, Tomoyo Taniguchi, Seiki Kobayashi, Hajime Hisaeda, Shinjiro Hamano

Affiliation number 1 for the first and last authors is incorrect. The correct affiliation is: Department of Parasitology, Institute of Tropical Medicine (NEKKEN), Nagasaki University, Nagasaki, Japan. 

